# Supplementary material for: Military Family-Centred Resilience-Building Programming Across the Deployment Cycle: A Scoping Review
Source: Int J Environ Res Public Health. 2024 Oct 18;21(10):1378. doi: 10.3390/ijerph21101378 (PMC11507616; doi:10.3390/ijerph21101378)
Supplement: Supplementary file 1 [file ijerph-21-01378-s001.zip › ijerph-3181418-supplementary.pdf]

Supplementary Materials

Initial Search Strategy

Table S1: Proposed Scoping Review Concepts

Concept 1 (Intervention)	Concept 2 (Program)	Concept 3 (Population)
resilience or resiliency program	program	military
resilience or resiliency service	service	veteran
resilience or resiliency intervention	training	military family member
overcoming adversity	education	military parent
self-regulation	session	child from military family
	webinar, seminar	youth from military family

NB: O from PICO often left out of title/abstract in searches; may be too narrow to include evaluation/impact – so will read for, not search for

Full Search Strategies

Ovid MEDLINE(R) ALL <1946 to December 19, 2023>

Date searched: December 20, 2023

Results: 380

1 Resilience, Psychological/ 8604

2 exp Program Evaluation/ or exp Program Development/ 106177

3 Curriculum/ or health education/ or health promotion/ or patient education as topic/ or self-evaluation programs/ or self-directed learning as topic/ or programmed instructions as topic/ 309522

4 1 and (2 or 3) 426

5 ((resilien* or self-regulation or overcom*-adversity) adj15 (program* or train* or teach* or learn* or educat* or session or sessions or webcast* or webinar* or website or app or apps or mobile-application* or seminar* or workbook* or podcast* or curricul* or bibliotherap* or skill*-development or develop*-skill*)).mp. 8853

6 4 or 5 9020

7 Military Personnel/ 45141

8 Veterans/ 22521

9 (Military or paramilitary or armed-force* or veteran* or armed-service* or servicewomen or servicemen or service-member* or air-personnel or defense-force* or defence-force* or service-personnel or army or navy or air- force or "marines" or sailor* or soldier* or infantryman or Civil-defense or Troops or ranger* or "medic" or active- duty or enlisted-personnel or reserve-personnel).mp. 170398

10 7 or 8 or 9 170398

11 6 and 10 380

Embase <1974 to 2023 December 19> (OVID Interface)

Date searched: December 20, 2023

Results: 442

1 psychological resilience/ 10130

2 program development/ 26264

3 exp program evaluation/ 36378

4 curriculum development/ 5786

5 curriculum/ or health education/ or health promotion/ or patient education/ or self-directed learning/ 434078

6 podcast/ or webcast/ or webinar/ 3092

7 1 and (2 or 3 or 4 or 5 or 6) 595

8 ((resilien* or self-regulation or overcom*-adversity) adj15 (program* or train* or teach* or learn* or educat* or session or sessions or webcast* or webinar* or website or app or apps or mobile-application* or seminar* or workbook* or podcast* or curricul* or bibliotherap* or skill*-development or develop*-skill*)).mp.10459

9 7 or 8 10753

10 military phenomena/ or air force/ or army/ or civil defense/ or military deployment/ or military research/ or military service/ or navy/ 30871

11 exp military personnel/ or veteran/ 41319

12 (Military or paramilitary or armed-force* or veteran* or armed-service* or servicewomen or servicemen or service-member* or air-personnel or defense-force* or defence-force* or service-personnel or army or navy or air- force or "marines" or sailor* or soldier* or infantryman or Civil-defense or Troops or ranger* or "medic" or active- duty or enlisted-personnel or reserve-personnel).mp. 184204

13 10 or 11 or 12 184204

14 9 and 13 442

APA PsycInfo <1806 to December Week 1 2023> (OVID interface)

Date searched: December 20, 2023

Results: 594

1 "resilience (psychological)"/ 22396

2 program evaluation/ or exp program development/ 23543

3 curriculum development/ or curriculum/ or educational program planning/39005

4 health education/15104

5 training/ 28313

6 1 and (2 or 3 or 4 or 5) 442

7 ((resilien* or self-regulation or overcom*-adversity) adj15 (program* or train* or teach* or learn* or educat* or session or sessions or webcast* or webinar* or website or app or apps or mobile-application* or seminar* or workbook* or podcast* or curricul* or bibliotherap* or skill*-development or develop*-skill*)).mp. 13884

8 6 or 7 13936

9 exp military personnel/ or military families/ 36063

10 military veterans/16866

11 (Military or paramilitary or armed-force* or veteran* or armed-service* or servicewomen or servicemen or service-member* or air-personnel or defense-force* or defence-force* or service-personnel or army or navy or air- force or "marines" or sailor* or soldier* or infantryman or Civil-defense or Troops or ranger* or "medic" or active- duty or enlisted-personnel or reserve-personnel).mp. 75807

12 9 or 10 or 11 76002

13 8 and 12 594

All of the following were searched using an EBSCOhost interface:

CINAHL Plus with Full text (262 results)

Military & Government Collection (265 results)

Health Source: Nursing /Academic Edition (133 results)

Education Research Complete (97 results)

ERIC (80 results)

Open Dissertations (53 results)

Criminal Justice Abstracts (29 results)

Child Development & Adolescent Studies (24 results)

Date searched: December 20, 2023

Deselect: Apply equivalent subjects and Apply equivalent words

(TI((resilien* or self-regulation or overcom*-adversity) N15 (program* or train* or teach* or learn* or educat* or session or sessions or webcast* or webinar* or website or app or apps or mobile-application* or seminar* or workbook* or podcast* or curricul* or bibliotherap* or skill*-development or develop*-skill*)) OR AB((resilien* or self-regulation or overcom*-adversity) N15 (program* or train* or teach* or learn* or educat* or session or sessions or webcast* or webinar* or website or app or apps or mobile-application* or seminar* or workbook* or

podcast* or curricul* or bibliotherap* or skill*-development or develop*-skill*)) OR SU((resilien* or self-regulation or overcom*-adversity) N15 (program* or train* or teach* or learn* or educat* or session or sessions or webcast* or webinar* or website or app or apps or mobile-application* or seminar* or workbook* or podcast* or curricul* or bibliotherap* or skill*-development or develop*-skill*))) AND (TI(Military OR paramilitary OR armed-force* or veteran* OR armed-service* OR servicewomen OR servicemen OR service-member* OR air-personnel OR defense-force* or defence-force* OR service-personnel OR army OR navy OR air-force OR "marines" OR sailor* or soldier* or infantryman or Civil-defense or Troops or ranger* or "medic" or active-duty or enlisted-personnel or reserve-personnel) OR AB(Military OR paramilitary OR armed-force* or veteran* OR armed-service* OR servicewomen OR servicemen OR service-member* OR air-personnel OR defense-force* or defence-force* OR service-personnel OR army OR navy OR air-force OR "marines" OR sailor* or soldier* or infantryman or Civil-defense or Troops or ranger* or "medic" or active-duty or enlisted-personnel or reserve-personnel) or SU(Military OR paramilitary OR armed-force* or veteran* OR armed-service* OR servicewomen OR servicemen OR service-member* OR air-personnel OR defense-force* or defence-force* OR service-personnel OR army OR navy OR air-force OR "marines" OR sailor* or soldier* or infantryman or Civil-defense or Troops or ranger* or "medic" or active-duty or enlisted-personnel or reserve- personnel))

Social Services Abstracts (Proquest interface - Command line searching)

Date searched: December 20, 2023

Results: 53

noft((resilien* or self-regulation or overcom*-adversity) NEAR/15 (program or programme or programming or train OR training or teach or teaching or teacher or learn or learning or educate or educating or education or educational or session or sessions or webcast or webinar or website or app or apps or mobile-application or seminar or workbook or podcast or curriculum or curricular or bibliotherapy or skill-development or skills-development or develop*-skills or developing-skills or developed-skill)) AND noft(Military or paramilitary or armed-force or veteran or armed-service or servicewomen or servicemen or service-member or air-personnel or defense-force or defence-force or service-personnel or army or navy or air-force or "marines" or sailor or soldier or infantryman or Civil-defense or Troops or ranger* or "medic" or active-duty or enlisted-personnel or reserve-personnel)

Table S2. Data Extraction Tool Draft.

**Authors**	**Year**	**Citation**	**Study Location**	**Setting**	Empirical<br>Review<br>Commentary, other<br>**Study Type**
M: Military members, Veterans<br>F: Family members<br>P: Providers, Deciders, Policy-makers<br>B: Both Military/Veteran $ families<br>**Population** **	Age<br>Gender/sex<br>Disability, other<br>**Population Demographics**	M: Military members, Veterans<br>F: Family members<br>P: Providers, Deciders, Policymakers<br>**# Population**	C: Clinician/professional<br>P: Peer (lived experience)<br>L: Lay person<br>**Provider**	P-P: Provider to person<br>T-T: Train the trainer<br>**Intervention Type**	P-D: Pre-deployment<br>AD: Active duty<br>T: Transition back to civilian/reintegration<br>**Intervention Timing**
**Objectives**	Design<br>Methods<br>Analysis<br>**Methods-QUAN**	Design<br>Approach<br>Analysis<br>**Methods-QUAL**	*Consider patterns of PTSD - Dr. Vermetten<br>Cross-sectional<br>Longitudinal - duration<br>Other<br>**Timeline**	Frequency, duration<br>One-on-one or in groups<br>Other characteristics<br>**Program/Intervention Descriptors**	P: Provision/therapy/TX<br>E: Education<br>T: Training<br>B: Both provision + education<br>**Style**
O: Online<br>I-P: In-person<br>H: Hybrid<br>**Delivery**	What instrument was used?<br>Self-report questionnaire<br>Observational questionnaire<br>Valid, Reliable instrument<br>Other characteristics<br>**Instruments**	What is measured?<br>PTSD/I<br>Resilience type (psychological, physical, social, collective or other)<br>Well-being or QoL<br>Other outcome(s), suicide prevention<br>**Outcome Variables**	**Explanatory Variables**	**Covariates**	When excluded, mark the excl. criterium not met<br>**Included/Excluded**
		What is measured?			
Methods<br>Rigour<br>Soundness, data trustworthiness<br>Analyses<br>**Comments on Research Quality**	What is the program reach?<br>Local<br>Regional<br>Jurisdictional or national<br>**Geographic Spread**	PTSD/I<br>Resilience type (psychological, physical, social, collective or other)<br>Well-being or QoL<br>Other outcome(s), suicide prevention<br>**Results**	Y: Yes, discussed<br>N: No, not discussed<br>**Adversity Y/N**	If adveristy is discussed:<br>B:Barrier<br>F:Facilitator<br>M: Mixed interpretation of adversity as a barrier or facilitator<br>**Adversity Thoughts**	**Additional Comments**

Table S3. Summary of the 32 Studies Included in This Review.

Article Identifier	Author (Year) Study Location	Program or Intervention Name	Objectives	Population	Participant Demographic	Design	Frequency (F)/ Duration (D)/Times Data Collected (TDC)	Mode[1]
1	Ashurst et al. (2020) Kentucky, USA	*Military Teen Adventure Camp* (MTAC)	(1) Highlight 2018 camp evaluation findings; (2) Explain a family-focused program model.	Teens, M, parents	**135**: 97 teenagers and 38 M where 13 military service member and spouses removed from analysis; Teens: almost 2/3 male, average age 15, 13-18 years; M: majority male, age unreported	Prospective, pre-post survey	F: daily<br>D: 4 days, 3 nights<br>TDC: baseline, completion	G
2	Bobrow et al. (2013) mainly California USA	*Coming Home Project* (CHP)	(1) Describe state frequency; (2) Determine impact on negative and positive states, short and longer term goal achievement.	M/V, adult family members, SP in 3 types of retreats	**347**: 175 M/V-adult family members at 1 of 4 military-family retreats, 126 SP 1 of 2 SP retreats, and 46 women-only in a retreat; M-family Retreats: just under ½ men, age 25-35 years, 1/3 married, more than ¾ had at least one child; SP Retreats: over ¾ women, age 28-65 years; Women-only Retreat: age 23-56 years; all had war-related deployment experience	Prospective, three-point repeated measures survey	F: daily<br>D: 4 days (V/M) or 5 days (V/M, Families)<br>TDC: baseline, completion, 30 to 60 days	G
3	Bui et al. (2018) Massachusetts USA	*Stress Management and Resiliency Training: Relaxation Response Resiliency Program*(SMART-3RP)	(1) Identify caregiver stressors; (2) Assess attitudes towards online program delivery.	Adult family members	**13**: civilian caregivers of V; majority female; average age 41 years	Prospective descriptive cross sectional survey	F: weekly 1.5 hours<br>Duration: 8 weeks<br>TDC: completion	G
4	Collinge et al. (2012) Vermont, Oregon USA	*Mission Reconnect* (MR)	(1) Determine instrument stability over time; (2) Describe perceived program impact.	V, spouses or partners	**86**: 43 V-civilian partner dyads; 27 Vermont, 16 Oregon; V: majority male; average age 34 years; Partner: majority female; average age 29 years; 2 missing data	Prospective mixed-methods design: pre-post survey and focus groups	F: weekly 3-4 days<br>D: 8 weeks<br>TDC: baseline, completion	C/F
5	Conover (2020) Texas, Alabama, Florida USA	*Tell Me A Story* (TMAS)	(1) Determine whether program increases resilience-related outcomes;	Children	**66**: 36 civilian parents, 30 children	Prospective pre-post survey	F: 1-day workshop (parents resourced	G

			(2) decreases problem behaviours.		Parents: age non-M average age 36.8, 25-46 years; M average age 37.7, 27-46 years; sex unreported;<br>Children: over ¾ female; average age 7.8, 6-10 years		to repeat at home 3-5 times per week)<br>D: 1 day<br>TDC: baseline, completion	
6	Dodge et al. (2018) Southeastern Michigan USA	*Strong Military Families* (SMF)	(1) Identify the program efficacy for reducing mental health symptomatology; (2) Better understand father perceptions of participation to increase future engagement.	M fathers	**14**: Fathers selected from larger sample of 107 parents previously deployed for at least 6 weeks and at least one parent had a child age 1-6 years; age 31-40 years; majority parents married	Prospective mixed-methods design: pre-post survey and pre-post interviews	F: weekly<br>D: 10 weeks<br>TDC: baseline, completion	G
7	Feinberg et al. (2020) USA	*Military Family Foundations* (MFF)	(1) Assess attrition; (2) Determine program impact on coparenting relationships, parental efficacy and depression in comparison with a control group; (3) Assess program impact on infant outcomes at six months of age.	M, spouses or partners, infants	**56**: heterosexual couples who were expecting their first child together; later their infants;<br>Couples: 6 fathers, 5 mothers previously had a child with another partner; majority married;<br>Mothers: age 29.7 years; Fathers: 31 years, infants 6 months;<br>Parents who are M: under 2/3 fathers, under 1/4 both parents, few mothers	Prospective pre-post randomised pilot control study	F: self-paced (online)<br>D: 6 months after birth plus initial participation (timeline variable)<br>TDC: baseline, completion	C/F
8	Garcia et al. (2015) USA	*Families Overcoming Under Stress* (FOCUS)	(1) Explore the training implementation in public schools; (2) Describe its implementation successes and challenges.	SP	**84**: 22 SP in year 1, 62 SP in year 2; social work interns; sex and age unreported;<br>Year 1: engaged with 202 students;<br>Year 2: engaged with 500 students; sex/gender, age, MF unreported	Cross-sectional mixed-methods design: two cross-sectional surveys and interviews	F: weekly D: 9 weeks<br>TDC: completion	G
9	Gewirtz et al. (2014) Minnesota USA	*After Deployment, Adaptive Parenting Tools*	(1) Describe extent of participation in activities–groups, online	M, parents	**75**: M and civilian parents with at least 1 child age 4-12 years;	Prospective descriptive pre-post	F: weekly 2 hours D: 14 weeks	G

		(ADAPT)	modules, home practice; (2) Determine program acceptability to families; (3) Explore association between baseline demographic factors and participation and acceptability.		about ½ mothers, ½ fathers in total; M (under 2/3): under ¾ fathers; Civilians (over 1/3): majority mothers; parent ages unreported	design on initial findings from a randomised effectiveness trial	TDC: baseline, completion	
10	Gewirtz et al. (2018) Midwestern State USA	*After Deployment, Adaptive Parenting Tools* (ADAPT)	Explore program effectiveness in comparison with a control group.	M, parents, children	**608**: M and civilian parents; 314 mothers, 294 fathers from 336 unique families who collectively have 336 children, average 2 children in the household, range of 1-6; less than ¼ single parents; Mothers: average age 36, 23-51 years; Fathers: average age 38, 23-58 years; Children: sex/gender unreported; 4-12 years	Prospective three-point repeated measures randomised controlled trial	F: weekly 2 hours D: 14 weeks TDC: baseline, 6 and 12 months	G
11	Julian et al. (2018a) 8 regions USA	*Strong Military Families* (SMF), Multifamily Group	(1) Examine the association between the program and parent behaviour and affect in the experimental group compared to a control home-based condition; (2) Examine reflective parenting as a mediator of the association	M/V, parents	**78**: M/V parents and civilian parents with at least one child age 7 years or younger, average age 4, 1-7 years; under 2/3 mothers, 3 grandparents; most parents age 22-40 years	Prospective pre-post single blind quasi-experimental study	F: weekly D: 10 weeks TDC: baseline, completion	G (Multi-family) C/F (Home-based)
12	Julian et al. (2018b) Michigan USA	*Strong Military Families* (SMF), Multi-family Group	Explore differences in parenting reflectivity among M mothers versus M fathers, and between M and civilian	M/V, parents	107: M/V parents and civilian parents with deployment history and with at least one child age 7 years or younger, average age 4, 1-7 years; under 2/3 mothers, 3 grandparents;	Prospective pre-post single blind quasi-experimental study	F: weekly D: 10-12 weeks DC: baseline, completion	G (Multi-family) C/F (Home-based)

			spouses/parenting part-ners in the experimental group compared to a control home-based con-dition		most parents age 22-40 years; about ½ M and others were spouses; 15 non-completers			
13	Kahn et al. (2016) San Diego, CA, Dal-las, TX, Fayetteville, NC, and New York, NY USA	*Kahn et al. (2016) San Diego, CA, Dal-las, TX, Fayette-ville, NC, and New York, NY USA*	(1) Evaluate the online program im-pact on mental health out-comes–depression, PTS, self-compassion, sleep, resilience, social support, and relationship sati-fac-tion; (2) Evaluate pro-gram use and satisfac-tion; (3) Explore the use of the program; (4) Ex-plore the presence of moral in jury in M.	V, spouses or partners	320: V-civilian partner dyads post 9/11 de-ployment with 181 Veterans and 139 civilian partners, 21 dual-V dyads; 3 same-sex dyads; V: majority male; Civilian partner: majority female; ages unreported; 4 non-completers	Prospective three-point repeated measures, four-arm randomised controlled trial	F: At participant dis-cretion<br>D: 16 weeks<br>TDC: baseline, half-way, completion	C/F
14	Knobloch et al. (2019) 44 locations USA	*REBOOT Com-bat Recovery*	Determine what charac-teristics predict im-proved physical health, mental health, and social health.	M/V, care- giv-ers, civilians	**254**: mixed population, 146 V who experi-enced combat trauma, 92 caregivers of V, 16 civilians with trauma or clinicians; over 2/3 married, rest single, dating, divorced, wid-owed or unreported; over ½ men; average age 44, 20-89 years	Prospective, pre-post survey	F: weekly<br>D: 12 weeks<br>TDC: 3 weeks, com-pletion	G
15	Le (2014) Colorado and Hawaii USA	*Mind Body Aware-ness Program* (MBA)	Describe program satis-faction.	Teens	**292**: 219 youth in Colorado and 73 youth in Hawaii; about ½ male; average age 15, 13-19 years	Cross-sectional sur-vey	F: daily (1 hour mind-fulness)<br>D: 4 days, 3 nights<br>TDC: completion	G

16	Lee & Kim (2023) Republic of Korea South Korea	*Thank You, Sorry, Love* (TSL)	Evaluate the TSL, a pi- lot project	Teens	**29**: first year high-school students transitioning to a new high school; ½ male; age 17 years; 23 from family with parent in army, 3 in Marines or Navy, 2 in Air Force; 2 unreported; 4–5 relocations and 2–3 school transfers	Prospective longitudinal design with 3 groups: TSL (experimental), Enhancing Resilience Program (comparison), control group; 9 or 10 teens assigned/group	F: 10 weekly sessions D: 10 weeks TDC: baseline, completion, two weeks after completion	G
17	Lester et al. (2012) 11 military installations in USA and Japan*	*Families Over-coming Under Stress (FOCUS)*	Evaluate FOCUS. Hypotheses: (1) Parents would report im-proved understanding of deployment and combat stress, improved family skills (emotional regulation, communication, family goal setting, management of stress reminders and triggers) and intrafamilial support, satisfaction with the program, and a likelihood of recommending the program to others. (2) Families would experience improved in-dividual psychological health outcomes and improved family functioning.	M, parents, children	1,615: 742 parents with 873 children from 488 unique families; 331 pre- and post-data and 466 parents during intervention: 300 non-active duty and 166 active duty with pre- and pos-assessment for at least 1 parent and 493 children from those families; Non-active duty: majority female and married, average age 34 years; Children: over ½ male, under 2/3 age 3-7 years, less than ¼ 8-10 years or 11 years or more	Retrospective design using secondary data analysis by com-paring those with and those without post data on severity of distress, participant scores or data to the null hypothesis (no change) or to community norms when available and those who improved with those who did not.	F: 8 sessions at family's convenience D: Parent and family sessions last 90 minutes and child sessions last 30-60 minutes, depending on the child's developmental level. TDC: Baseline, exit, and 1, 4 and 6 months after intervention	CF

18	Lester et al. (2013) 11 military installations in USA and Japan*	*Families Overcoming Under Stress* (FOCUS)	(1) Determine the relationships between distress and family relations, specifically emotional awareness and regulation; (2) Evaluate program impact pathways.	Parents, children	**280**: civilian parents who were primary caretakers of 505 children and the other parents are M;<br>Parents: sex/gender & age unreported; Children: under ¼ female, average age 7, 3- 17 years	Retrospective four-point repeated measures study	F: 8 sessions D: Unreported<br>TDC: baseline, completion, 1 month, 4 to 6 months	C/F and G
19	Lester et al. (2016) 15 military installations in USA and Ja- pan	*Families Overcoming Under Stress* (FOCUS)	Examine consistency over time of improvement in psychological health across both parents (civilian & military) and children, and in family adjustment & child coping.	M, parents, children	**7,309**: 3,499 parents with 3,810 children from 2,615 unique active duty families with at least 1 child 3-17<br>years of age with a M parent serving;<br>M parents (1,426): over 2/3 male, average age 34 years;<br>Civilian parents (2,073): majority female, average age 33 years;<br>Children: about ½ male, average age 7 years	Retrospective three-point repeated measures study	F: 8 sessions D: Unreported<br>TDC: baseline, completion, 2 months	C/F and G
20	Mikolas et al. (2021a) Canada	*Bounce Back and Thrive!* (BBT) & *Reaching In…*<br>*Reaching Out* (RIRO)	Describe service provider perspectives about the program training, specifically,<br>(i) their ability to model teachings and deliver the program,<br>(ii) program's appropriateness for M and Families, (iii) pro- gram's ability to ad- dress training gaps,	M, parent, SP, decision-makers	**24**: SP serving children age 0-8 years; MFRC: 8 social workers, early childcare workers;<br>Royal Canadian Chaplain Service/RCChS: 5 chaplains;<br>CAF transition unit: 1 M, 1 civilian parent;<br>Local health authority: 1 social worker; University-based researchers/clinician-scientists: 4 - nurse, occupational therapist, psychologist, social worker;<br>Leaders from MFRC and RCChS: 2; Master Trainer: 1	Cross-sectional focus groups and interviews	F: 10 2-hour sessions (BBT), 12 hours total (RIRO)<br>D: Approximately 10 weeks<br>TDC: completion	G

			(iv) program's prospects for fostering a culture of resilience.					
21	Mikolas et al. (2021b) Canada	*Bounce Back and Thrive!* (BBT) & *Reaching In… Reaching Out* (RIRO)	Describe the program (i) impact on building military parent well- being, (ii) facilitation of resilience-building, (iii) contextualisation for M and Families, (iv) support for MFRCs cultivating a culture of resilience.	M/V, par- ents	**9**: 5 civilian, 2 M, 2 V parents; over ¾ female, about ½ M/V, age unreported; about ½ married; Children birth-8 years: sex/gender unreported; over ¾ families with 1 child, rest either 2 or 3	Cross-sectional focus groups and interviews	F: weekly 3, 6 or 10 sessions (standard) D: Weekly TDC: completion	G
22	Oades-Sese et al. (2021) San Diego, CA USA	*Little Children, Big Challenges: General Resilience* (LCBC) a Sesame Street Program	Determine the effectiveness of the pro- gram in preschool classrooms in comparison with a control group.	Children, teachers	923: 766 children (few from military families) and 157 teachers from 62 preschool sites, under 2/3 Head Start Centres, less than ¼ either public preschools, community-based or Military Child Development Centres; Teachers: majority female; average age 42 years; Children in 159 class groups: over ½ female, average age 4, 3-5 years Classrooms: over 2/3 dual-language, under ¼ English-only	Prospective pre-post single blind Quasi-experimental cluster-randomised study	F: daily (5 sessions/ week) D: 12 weeks TDC: baseline, completion	G
23	Pinna et al. (2017) Minnesota USA	*After Deployment, Adaptive Parenting Tools* (ADAPT)	(1) Describe family engagement and satisfaction with the pro-gram adaptation; 2) Determine whether groups led by peer facilitators* yielded	M/V, parents	336: 207 parents in intervention group and 129 parents in services as usual group with 1 biological, adoptive, or stepchild; Parents: over ½ mothers, average age 36 years, majority married;	Prospective descriptive design with only the experimental group reported from a	F: weekly D: 13-14 2-hour sessions, 14 weeks TDC: weekly attendance, assignment	G

			better engagement and satisfaction; (3) Describe engagement and satisfaction differences between mothers and fathers.		Children: sex/gender unreported, age 4-12 years; average 2 children at home	randomised controlled trial; military-connected facilitators were randomly assigned to experimental or control groups	completion and satisfaction from outset	
24	Ruiz et al. (2020) Indiana USA	*Families Tackling Tough Times Together* (FT)	Describe (i) number of times program is accessed, (ii) information shared, (iii) knowledge gained.	SP, Decision-makers, adult family members	**72**: M families and civilian families; 60 SP, university students and 12 external partners in Facebook working groups; Roles: 25 staff, 19 faculty members, 6 clinical faculty, 5 graduate students, 4 administrators, 1 undergraduate student	Program development and implementation	F: weekly D: 10 weeks TDC: weekly from outset	G
25	Saltzman et al. (2016) 14 military installations in USA and Japan**	*Families Overcoming Under Stress* (FOCUS)	(1) Determine whether level of baseline parental dis- tress and symptom expression are predictive of response to treatment; (2) Deter- mine if the benefits of participating in the program are impacted directly or in- directly by improvements in key family resilience processes.	M, parents, children	**434**: 210 fathers, 210 mothers; 4 unreported; M parent: majority male, average age 34 years; Civilian parent: majority female, average age 33 years; Children: sex/gender, average age unreported, average 1.82, 1-5 children at home	Retrospective, three-point repeated measures study	F: weekly D: 8 weeks TDC: baseline, completion, 4 to 6 months	I, C/F,
26	Schachman et al. (2004) Midwest USA	*Baby Boot Camp* (BBC)	Determine program impact on maternal role adaptation in comparison with a control group.	Civilian mothers-to- be	**91**: 44 pregnant women in BBC group and 47 pregnant women in routine care group, all at 32-37 weeks gestation and married to M spouse; about ¾ were younger than 22 years, ranging 18-28 years	Randomised controlled trial	F: weekly 3-hours D: 4 weeks TDC: baseline, completion, 6 weeks postpartum	G

27	Sherman et al. (2011) Oklahoma USA	*Reaching out to Educate and Assist Caring, Healthy Families* (REACH), a Multifamily Group model	(1) Describe participation, retention, and satisfaction in the program; (2) Assess program fidelity.	V, spouses or partners	**232**: 116 Veteran-family member dyads, majority family members were spouses; ½ V had PTS, other ½ affective disorder; V: majority male, average age 50 years or more, 20-60+ years, majority married	Prospective, three-point repeated measures study	F: weekly to monthly (varies by program phase)<br>D: 9 months<br>TDC: phase completion: Phase I–4 weeks, Phase II–6 weeks, Phase III–6 months (total 8.5 months)	C/F and G, de- pending on the phase
28	Sylvia et al. 2022 Massachusetts USA	*Stress Management and Resilience Training Relaxation Response Program (SMART-3RP)*	Examine the acceptability and explore the effectiveness of a mind-body stress management program, and whether it was more effective delivered in person or online.	Caregivers, adult family members, possibly SPs	**55**: Family members, defined broadly as caregivers of Veterans who were 23 V, 11 M, 3 reservists, 17 other;<br>Caregivers: majority female, average age nearly 40 years;<br>natural only or some paid SPs unreported	Prospective pre-post design and qualitative feedback	F: weekly<br>D: 90-minute sessions, 6 weeks<br>TDC: baseline, completion	I
29	Weis et al. (2017) Texas USA	*Mentors Offering Maternal Support* (MOMS)	Determine the efficacy of the program for prenatal anxiety, self-esteem, depression in comparison to routine care.	M, civilian mothers-to- be	**367:** 187 pregnant women in intervention group, 180 pregnant women in routine care group, average gestational age 9 weeks, majority married, average age 29 years, over 1/3 pregnant with their first child, about ¼ experienced partner deployment during pregnancy, and the majority were M	Randomized controlled trial with three-point repeated measures	F: every other week 8 1-hour sessions<br>D: 16 weeks<br>TDC: baseline, 2nd or 3rd trimester, 21-30 weeks gestation	G
30	Weston et al. (2021) USA	*Military Teen Ambassador's* (MTA)	Describe program impact on youth knowledge of resilience and reintegration, self-perceived personal growth and skill development, and community needs awareness.	Teens	**91**: teen ambassadors, including 8 peer facilitators; about ½ female, 7 sex/gender un- reported, age 14-18 years, with most either 15 or 17 years	Prospective, three-point repeated measures sur- vey	F: daily D: 3 days<br>TDC: baseline, completion, 6 months	G

31	Wilson et al. (2011) Indiana USA	Passport To-wards Success (PTS)	(1) Describe stressor experiences associated with military pa-rental deployment and reunion – base-line; (2) Assess pro-gram fidelity; (3) Describe program use-fulness from children's perceptions, specifically gaining new ideas about skills that facilitate resilience.	M, civilian parents, children, teens	161: 77 male children, 84 female children from 88 families;<br>M parents: majority fathers or stepfathers;<br>Civilian parents: majority mothers or stepmothers;<br>Parental age unreported;<br>2/3 mothers or stepmothers completed surveys;<br>Children: average age 3-6 years or 7-11 years each under ½), 12-17 years under ¼; average 2.5, 1-6 children at home with M parent; children 9+ years completed pre-, post-tests	Prospective, pre-post survey	F: 1 day<br>D: 2.5-4 hours (depending on setting)<br>TDC: baseline, completion	G
32	Van Breda (1999) USA	Separation Resilience Seminar (SRS)	Evaluate program implementation on satisfaction and effectiveness.	M, spouses or partners	34: 18 M-civilian spouse dyads;<br>Sex/gender unreported, average age 30, 24-37 years, all married, majority with 1 or more children<br>Children: average age of oldest child 6 years	Prospective, three-point re-peated measures sur-vey with 2 groups post hoc	F: 1 day<br>D: 1 day<br>TDC: baseline, completion, 2 months	G

1: The program was provided to either individual participants (I), to individual couples or families (C/F), or to individuals, couples or families in groups (G); M: Military serving, inactive members or reservists; V: Veteran members; SP: service providers; MFRC: Military Family Resource Centre; *: California, North Carolina, Hawaii, Virginia, Mississippi, and Washington State (USA), and Okinawa (Japan) **: 14 military installations in in California (four sites), North Carolina, Hawaii, Virginia (two sites), Mississippi, Washington State (USA), and Okinawa (Japan).

Table S4. Summary of Results and Evidence Levels for the 32 Studies Included in This Review .

Article Identifier	Deployment Cycle Timing	Results	Evidence Level[1]
1	Reintegration, after an extended deploy-ment- related ab-sence	A randomly chosen participating teen in the camp would have greater post-program resilience 56% of the time. Teens scored at the 60th to 70th percentile and exceeded other campers, on problem-solving and connectedness. Teens scored above and below the 50th percentile on Family Citizenship. A randomly chosen participating M in the camp would have greater post-program resilience 62% of the time. Anecdotal reports suggest improved family communication due to distraction-free outdoor activities, which partici-pants most often ascribed to no ac-cess to cell phones and other electronic devices.	II
2	Active-duty or rein-tegration	There was a lower percent of married couples in the military-family retreats compared to other family programs. Positive change noted 4 to 8 weeks after intervention in 13 of 15 state items, yet no sustained improvement in "Able to care for self" and "Hopeless" states. Highest ratings reported were on feeling less alone and gratitude for community. Least improved rating was in decreasing physical pain. In the military- family retreats, reductions in stress and isolation, as well as improvements in relaxation and hope, for all retreat participants; ratings of positively worded items such as "energized" and "supported" significantly increased over the course of the retreats, whereas ratings of negatively worded items such as "stressed" and "emotionally numb" significantly de-creased over the course of the retreat. Goal achievement ratings in these retreats were high 4.57 (SD 0.29) on a scale of 1 to 5, rang-ing from 3.61 to 4.95. In female-only retreats, goal achievement was high 4.41 (SD 0.25), ranging from 3.68 to 4.70 in gratitude for community and learning from others' experiences, and least in decreasing physical pain and in finding meaning in the difficult expe-riences they had. In the SP retreats, goal achievement was fairly high 4.17 (SD 0.24), ranging from 3.62 to 4.51 with highest in feeling not alone and gratitude for community and least in decreasing physical pain. Stigma appeared to be reduced. Participants reported being more open to accessing psychological and other resources to continue on their journeys toward reconnecting with self, their peers, their families, and communities. Variable feedback: Female-only participants appreciated the opportunity to share in a safe and compassionate setting. SPs accepted sharing about similar lived experiences and connecting with others and self. Some family participants wanted more small-group activities to further discuss experiences and reconnect with their spouse. Cohort-specific groups were generally accepted, but some would separate V with their respective family members from active duty M and from wounded warriors to distinctly honour those who were exposed to trauma and those who were not. Similarly, SPs suggested profes-sion-specific group activities, less talking and more quiet time, and a social outing to talk rather than during meditation.	II

3	Unreported	Online portability was well accepted, yet Mp3 was suggested over phone to limit distractions. Compliance/accountability may be reduced online compared to in person due to failing to prioritise own needs. 1/13 not comfortable with technology. Intervention seen as beneficial for physical effects of stress, cognitive reappraisal, mindfulness, specific language/content for military caregivers, communication skills, setting practical/achievable goals, and setting healthy boundaries; time invested could be a maximum of 45 minutes because of unpredictable/chaotic schedules; rigid adherence would not work.	III
4	Reintegration	Fidelity exceeded the minimum instructions. Participants averaged over six times/ week using one or more of the mind/body exercises and more than 2.5 times using massage. Areas massaged were shoulders (75%), neck (72%), back (68%), head (36%), and feet (27%). Veterans reported highly significant decreased physical pain, physical tension, irritability, anxiety/worry, and depression post-massage. Substantial risk of PTS was identified in both V and partners, and both showed modest improvements at both follow-ups. Both V and partners showed significant decreased depression symptoms from the mild range to the minimal range at follow-up. Partners showed significant reductions in perceived stress, and both partners and V had significant improvements in self-compassion at first follow-up and a trend at second follow-up. There were no significant changes on the Compassionate Love Scale, although over half of the subjects improved their scores at both follow- ups. No changes noted in QoL. Participants reported practicing the exercises at home, at work, and in their vehicles. Also, the program may be able to reach people who are geographically isolated from services as well as people who are reluctant to use mental health services. A noted weakness is that massage may be too intimate for participants to invite a massage partner other than one's spouse/partner.	II
5	Pre-deployment or active-duty/post-deployment	Girls decreased problem behaviour and boys increased. Families who experienced deployment during the study period reported increased problem behaviours compared to nondeployed families with a very large effect size. Girls who experienced parental deployment had a greater decrease in problems than girls with nondeployed parents with a very large effect size. Overall, problem behaviours increased. Those who did not experience parental deployment decreased internalizing problem behaviour with a large effect size. No difference found for externalizing behaviours.	II
6	Active-duty	Pre-interviews, fathers wanted learning to be on parenting, military families, and communication, and connection to be on relating to others with similar lived experience. Also, fear of committing was noted due to busy schedules and providing family support. Post-interviews, fathers gained better understanding of the parent-child relationship as a whole and tools to communicate and interact with the family to facilitate healthy relationships. They also reported a sense of community through sharing with others who had similar lived experience, learning from and connecting with other families in the program. Parenting was defined as being able to see the parent-child relationship from a different perspective. Limitations: One participant fit the criteria of PTS after, but not before the program. This participant was removed from the analysis comparing symptomatic versus non-symptomatic parents due to concerns	II

		for maintaining participant confidentiality. Also, statis- tical analyses revealed significant decreases in self-reported posttraumatic stress and reduction trend in depression.	
7	Pre-deployment then active-duty	Overall sample representative of total service member population. Average gestational weeks were 24.4 at time of recruitment. Depression scores lowered in the intervention group, but efficacy showed no difference in groups. Intervention couples completed less than 4/8 modules (3.93). Compared with control parents, intervention parents reported that infants showed less sadness, and mothers' reported that infants were more shootable. There was also a trend toward reduced infant distress. Attrition: Three couples were lost at Time 2 due to study closure before they responded. Fathers who attritted had higher household incomes than completers. Email messages were used to minimise attrition.	I
8	Pre-deployment, active-duty/post- deployment, reintegration	Overall training satisfaction was high. Findings on training the interns show some promise. All learning modules were rated high. Communication and Goal Setting were rated the highest (4.59 and 4.13 on 5, respectively). Readiness to deliver the emotional regulation group and communication group were fairly high (7.78/10, 7.4/10 respectively). Lowest readiness was Deployment Reminders (3.91/5). Only 13.6% of the interns viewed the supervision/consultation with faculty/instructors as helpful or very helpful. Suggestions for improvement included longer standing mentorship with co-facilitation of a group in the schools to start the intern off, instead of a conference call, then staying in touch with the mentor as needed. Also, the training timing; before academic year start, over 7 weeks not 2 days, in shorter after-school sessions, because learning is minimised from fatigue. One concern raised related to 'time to get into deeper issues when students talked about … death of a family member'. The Feelings Thermometer was the most used tool by some interns. This tool may be more useful with girls who were more able to express their emotions effectively and readily share how they are feeling than boys. 17 interns shared reduced anxiety in students. Interactions improved after group 4 or 5, and new friendships developed. Supportive administration was helpful with implementation. Conversely, a disinterested principal prevented student participation for 1 intern. Challenges in schools included not easily locating military children, so having a check-box in the system to notate military families would facilitate invitations. Also, lack of reserved time and competing priorities with many other groups to run, so not all interns could fit this group in. Lack of budget to acquire material to run activities was another barrier. Also, no designated space impeded implementation. Getting consent to participate and conflict with faculty-intern when trying to pull students from class during class hours were additional challenges.	III
9	Reintegration	78% of families invited to participate in the intervention came to at least one weekly 2-hour session. Most parents 92% only attended one or more sessions and parents completed 0 to 12 on 13 home practice assignments. The most used online tools were summaries (33%), knowledge checks (32%), and videos (30%). Among the least used online tools was mindfulness. Parents questioned how mindfulness was relevant to parenting, which may explain the limited use of this tool. Higher educated families accessed	II

		videos more frequently. Heightened attendance correlated with greater use of online tools (hope practice assignments). High satisfaction with 14 sessions (nearly 4/4), low to moderate satisfaction with home practice assignments/online tools (1.72 to 2.29/4). Only home practice satisfaction was a significant predictor of home practice completion, and general satisfaction and positive group experience were not. Data from the first two cohorts of ADAPT intervention participants suggests that the program is both feasible and acceptable. ADAPT may have been perceived as community-based parent education program, and a source of peer social support, rather than MH support/service. Families suggested that they particularly enjoy ADAPT because the tools provided in the program are very consistent with what they value in military life (i.e., structure, clarity, routines, protocols, and the like). ADAPT may be equally acceptable and relevant to both genders. Social desirability may have influenced too due to leaders and officers who strongly endorsed ADAPT.	
10	Active-duty	Findings indicated that ADAPT ITT was associated with small to moderate effect sizes for improved parenting practices. ADAPT was significantly associated with increases in effective parenting 1-year later relative to controls. The improved parenting practices associated with the ADAPT ITT intervention in turn predicted a moderate effect in childhood adjustment, explaining 2% of the variance in this construct. Couples who were married longer showed decreases in effective parenting.	I
11	Active-duty or reintegration	Relative to the control condition, Multifamily Group participants showed promise for improvements in aspects of positive parenting (Emotional Responsivity, Positive Affect), but no decreases in negative parenting. The interactive nature of the group may have helped parents to more deeply understand and apply the intervention's psychoeducational material, resulting in greater improvement in their positive parenting skills. Intervention effects appeared to be specific to aspects of parenting related to positive emotionality and enthusiasm, with no apparent effects on aspects of parenting related to negative or flat emotionality or instrumental support. The intervention appeared to work equally well for all military families with young children and may be beneficial as prevention even in families that do not show parenting difficulties. The efficacy of the Multifamily Group did not appear to depend on parent risk level or preintervention parent behaviour and affect. No intervention effects were detected for parents' Behavioral Responsiveness, Withdrawal/ Depression, or Irritability/Anger in interaction with their young children. Barriers to participation: Geographic dispersion of families and the distance required to attend, timing of the group not fitting the family schedule, enrolling in the study before the start of a Multifamily Group in their region, and/or preference for the at- home written materials.	II
12	Active-duty or reintegration	Preliminary analyses revealed that Multifamily Group participants scored marginally higher in reflective parenting than the control group at baseline. Baseline parenting reflectivity scores were significantly correlated with post-intervention parenting reflectivity. Hierarchical regression analyses revealed that intervention condition explained a significant amount of variance in parenting reflectivity scores. There was also a marginally nonsignificant association between baseline and post-intervention parenting reflectivity	II

		scores for the control group. In the Multi- family Group, more parents changed from "Less Reflective" to "More Reflective" (42.4%), and 3 times more participants in the control group changed from "More Reflective" to "Less Reflective" (34.5%) than the experimental group (11.5%).	
13	Reintegration	Hypothesis 1 was partially supported insofar as Mission Reconnect that showed significant mental health improvements compared to other control conditions of the study, except on sleep quality, lack of movement in dyadic improvements, and perceived social support. The other three hypotheses were supported, indicating that this program was generally effective, with greater effect on V. The V and their partners used some aspect of Mission Reconnect at least 2.5 hours per week, 20 x/week. Average use of non massage practices was 17 x/week, and average massages was 1.4/week for both Veterans and partners. The most frequently used cluster was Connecting with Yourself, with morning gratitude (2.6 x/week) being the most popular, followed by morning greeting (2.5 x/week). Twice as much time was spent in the Connecting with Quiet and more than that in the Connecting with Your Partner segments. Standard deviations for the use of various exercises varied widely, indicating that different participants used this online intervention with highly variable frequency. Dyads both spent over half their intervention time in activities that enhanced connecting with their partner. Non-Mission Reconnect participants used many techniques such as prescription & non-prescription drugs, sex, meditation, exercise to support their mental health. V averaged 3.6 activities/week while partners averaged 3.9 activities/week. Significant differences were found in the Mission Reconnect arm compared to the other arms, especially in stress and sleep quality during the first 8 weeks, and even more significant differences were found at 16 weeks. At 8 weeks, no significant differences between the two non-Mission Reconnect arms were found. Partners differed significantly from V on program outcomes. V used the program less over time, and the Mission Reconnect-only arm showed changes in same areas for partners and V, but effects were lower for partners. All participants were highly satisfied with the program.	I
14	Active-duty/post-deployment or reintegration	Overall, this program was associated with improvements in the domains of mental, and social health, and offered promise for peer-led pro- grams in the future. Improvement was observed in sleep disturbance, anxiety and depressive symptoms, and social participation. No improvement was observed in physical function or pain intensity. Participants with combat or civilian trauma reported greater anxiety and reduced social participation than caregivers. Males reported worse physical function scores, pain, sleep disturbance, and social participation than women. Improvement did not vary by role, gender, age, # of sessions attended, or graduate status. V reported poorer physical functioning, pain interference and intensity, sleep disturbance, and anxiety and depressive symptoms. Pain interference, intensity, sleep disturbance, and depressive symptoms were higher among those with more months of combat deployment. When interaction effects were tested, V re- ported more improvement than active duty personnel, indicating that the REBOOT program was more effective for V than AD personnel in terms of anxiety symptoms.	II

15	Unreported	Youth seemed to enjoy the program and experienced some perceived benefit from the mindfulness activities. In Colorado, five themes emerged: (1) being in nature and outdoors, (2) connection and acceptance, (3) cooperation, (4) fun and enjoyment, and (5) learning new skills to deal with stress. Survey responses suggested that the youth found the program helped them to better relax. Counsellors shared how the program helped transform their perceptions of military youth. They reported having a new appreciation for their circumstances and were moved by how vulnerable and open the youth were. They also believed that being in nature helped the process of camp and the bonding that occurred between members. In Hawaii, further responses were recorded about which activities the youth enjoyed the most: surfing, ropes course, free time, mindfulness, and paddle boarding (in that order). Mindfulness was ranked first as the activity that helped them form the strongest bonds.	III
16	Active-duty	Suggestive results due to the small sample size and missing details about data analysis. Potential intra-participant differences were found at both the post-intervention and follow-up scores in resilience, stress, depression, and family function. School adaptation showed no difference at follow-up. The comparison group received the Enhancing Resilience Program (Joe et al., 2015), including "understanding myself" (sessions 1–3), "improving problem-solving capacity" (sessions 4–6), "improving social skills" (sessions 7–8), and "planning for the future" (sessions 9–10). One male dropped out of the experimental group prior to starting intervention.	II
17	Active-duty	Family members reported high levels of satisfaction with the program and positive impact on parent–child measures. Psychological distress levels were elevated for service members, civilian parents, and children at program entry compared with community norms. Boys and girls also entered with higher scores compared to community norms. Change scores showed significant improvements across all measures for service member and civilian parents and their children (P<.001). 363 parents rated family improvements (emotional regulation, understanding combat stress and parent-child stress reactions), program satisfaction and high willingness to recommend the program to other families at about 6 out of 7 rating, similarly for both active duty and non-active duty parents. In children, reported improvements were found in difficulties and prosocial behaviour except for children 8 to 10 years of age, and in symptom reduction. Reductions in the prevalence of children with clinically significant symptoms over time were all significant (P<.001). About 1/3 of individuals entering the program were also referred to other social support and mental health providers, indicating the potential for selective prevention as a gateway to other services when needed. No baseline differences were found between active duty and non-active duty family functioning and distress, but both groups were significantly more distressed than were community norms. More non-active duty parents presented high PTS signs, where 33.7% (n=150) were above the cutoff for elevated posttraumatic stress symptoms at intake compared with 23.3% (n=69) of active duty parents. Attrition: 2/3 or 331 families completed the intervention; 89 (18.2%) attritted because of relocation or deployment, 42 (8.6%) reported their family was too busy to complete the program, 13 (2.7%) reported they no longer needed services, and 13 (2.7%) discontinued for unspecified ''other''	II

		reasons. Families and parents who completed the intervention were more likely to be self-referred, less distressed, and older than non-completers. Children strengths/difficulties who completed the intervention did not differ from non-completers.	
18	Active duty	Civilian parents participated. The FOCUS program appears to be associated with reduced distress in military children. Greater dosage or number of sessions attended predicted greater improvements. Also, more distressed children and family members benefitted the most. Younger children and boys were more likely to have distress than older children or girls. Greater distress in the military parent predicted the number of visits attended during the FOCUS intervention. Positive change in the Family Assessment Device (FAD) also predicted reduced child dis-tress. Further, the number of visits had an indirect effect on stress, where positive change in FAD medi-ated the relationship between number of sessions attended and reduced child distress. Finally, an analysis of individual FAD compo-nents revealed that the affective involvement subscale had the most influential effect on predicting changes in child distress by ex-plaining 11% of variance. Additional effects were as follows: problem-solving and communication explained 9% of variance respec-tively, roles 7% of variance), affective responsiveness 3% of variance), and behavioural control none of variance.	II
19	Active duty	Overall, the FOCUS program appeared to be associated with reductions in psychological difficulties (e.g., anxiety, depression) and improvements in family functioning and positive coping, albeit to varying degrees for each. The follow-up analyses indicated that these improvements were durable at 1-month and 6-month follow-ups. Of note, M parents reported lower levels of anxiety and depression than civilian parents, where 23% of M and civilian parents reported clinically meaningful anxiety symptoms, and 27% of M parents and 20% of civilian parents showed significant levels of depression. 26% of SM and 31% of civilian parents reported PTS scores that were clinically significant. Civilian parents reported lower levels of unhealthy family functioning relative to M parents, and nearly 50% of M and 43% of civilian parents reported unhealthy family functioning. 35% of boys and 25% of girls scored high in terms of "total difficulties". At baseline, 19% of boys and 12% of girls had high difficulties with prosocial behaviours, where boys had significantly higher total difficulties than girls. On the other hand, girls reported significantly greater anxiety than boys, but both 14.3% of boys and 14.7% of girls ages 8 to 17 years reported clinically elevated levels of anxiety. Mean scores on cognitive restruc-turing and problem-solving were similar for boys and girls. Girls scored higher than boys on emotional regulation and social support. Parental mean scores for anxiety symptoms decreased at each follow up. M parents showed the same trends, but the mean differ-ences were lower than those found with the total group, and civilian parents showed same trend, but their mean anxiety score changes were higher than the M group and total group. Parents showed a reduction in mean depression scores. How-ever, depres-sion symptoms seem to have increased slightly at first follow-up, then decreased at second follow-up. The change observed in mean scores was lower for M parents than civilian parents. The percentage of clinically meaningful depression and anxiety scores de-creased from 23% to 11% of the sample, a pattern which remained consistent at follow-ups. Both M and civilian parents reported	II

		significant de-creases in PTS symptoms from intake to post-intervention. All parents reported a decrease in unhealthy family functioning and reduced odds of meeting cut off for unhealthy functioning. In children, reductions in total difficulties were found at both follow-up assessments, and smaller but significant improvements were found at both follow-up assessments. Interaction regression models revealed that total difficulties and prosocial behaviours improved more for boys than girls. Also, children's anxiety symptoms showed improvement. For children ages 8 years or more, prevalence of clinically significant anxiety decreased from 14.5% to 11.8% at post-program, with reduced odds of clinically significant anxiety as well. Child-reported coping skills variably improved. Differences between families who partially completed compared to those who completed the program revealed that symptom reduction was similar at intake, then became larger, but not significant at final follow-up.	
20	Active-duty or reintegration	Overall, service providers (SP) indicated that they felt RIRO/BBT could fill a need in military communities, that it was useful, helpful, and could be effective. Four themes emerged: (1) the training enhanced SP's abilities to model resilience and deliver RIRO and BBT; (2) RIRO & BBT pro- grams would be useful for families and communities; (3) SP believed that these programs could assist in developing resilient communities; (4) the program could be adapted for military populations. Further, SP expressed that the program equipped them with useful skills and tools to use in service of their communities, could fill current needs in Military Family Resources Centres, cultivated a common language for talking about resilience, and noted that the training improved themselves and their own families.	III
21	Active-duty or reintegration	Overall, participants felt that this program was helpful in facilitating resilience-building yet could be better contextualized for military families. Partnerships with MFRCs could be useful for promoting resilience within the community. Four themes with respective sub-themes emerged: (1) Program impact on parent resilience: Participants believed that the BBT helped them see that resilience (i) is a learned skill, (ii) requires reflection and awareness, and (iii) requires practice. (2) Program impact on military family resilience. Participants described the BBT as (i) supporting the development of collective family resilience, (ii) requiring them to reflect on parenting practices, and (iii) required a collective understanding and practice of resilience skills (e.g., practicing resilience skills at home). (3) Program feedback: Participants described BBT as (i) supporting military family resilience-building, (ii) promoting peer connection during BBT, which was felt to be essential, (iii) that the program content was valuable to them, (iv) that BBT was helpful but still required some contextualization for military families such as more father representation, and (v) that BBT provided a flexible program delivery that was well-organized and "flowed" well. (4) MFRCs as Community Hubs: Participants stated that (i) having BBT provided at a local MFRC would be helpful, given that MFRCs are well-established and trusted by the community, (ii) that the BBT program may be well-positioned to address a gap for spouses with its psychoeducational, peer- supportive, and specific training	III

		approach, and (iii) that MFRCs' existing partnerships in the community may facilitate BBT's integration into Canadian Armed Forces programming.	
22	Unreported	All study hypotheses were supported, with the exception of Hypothesis 2. Overall, the program appeared to improve children's emotion vocabulary and had a small, positive effect on their socio-emotional behaviours, except for social problem-solving, with no significant effect. The teachers who helped deliver this program to the children also found the program useful and easy to implement. Hypothesis 1: Positive gains in emotion vocabulary were observed in the experimental condition and medium gains in the control condition. Hypothesis 2: The effect of the program on improving behaviour in social problem solving was not significant. Hypothesis 3: For each problem behaviour, small but significant declines were observed, whereas small and non-significant changes were observed in the control condition. Further, positive behaviours increased in the experimental condition, but either very little or not significantly in the control condition. Hypothesis 4: In the experimental condition, attachment increased a moderate amount and teacher conflict was reduced a small but significant amount. How- ever, in the control condition, attachment saw no significant gain and teacher conflict increased a small but significant amount. Hypothesis 5: Teachers indicated that in both conditions the intervention was appealing, useful, and easy to implement.	I
23	Active-duty/post-deployment or reintegration	Overall, the ADAPT program suggested promotion of participant engagement and satisfaction. Whether it resulted in statistically significant improvements in the pertinent resilience-related variables of interest, however, remained questionable. Engagement: At least one parent attended 1 or more sessions in about ¾ of all sessions. Attendance did not differ significantly between mothers and fathers, after accounting for controls. Families who attended at least 1 session completed an average 63% of the home practice assignments. Satisfaction: Satisfaction was high overall, with mean scores ranging from 3.37 to 3.59 on 4 across all sessions. Satisfaction scores were also similarly high between M and non-M groups and between mothers and fathers. Positive Group Experiences were high, with mean scores across sessions ranging from 3.31-3.50 for individual sessions and 3.39 across all sessions. Mothers reported more positive group experiences than fathers after accounting for controls. Home practice satisfaction was also consistently high, yet, again, home practice satisfaction did not significantly differ between M and non-M groups, after accounting for controls. No significant gender differences were observed for home practice satisfaction, either. Attrition: Attendance was significantly higher in sessions led by peer facilitators. Completion did not differ between M groups and non-M groups, after accounting for relationship status. Home practice completion also did not significantly differ between mothers and fathers, after accounting for controls. Older parents completed fewer home practice assignments and reported lower satisfaction.	II
24	Unreported	On average, 34% of the members were active in any given week, meaning that they viewed or reacted to one or more posts. 1,363 members from 25 countries have joined the Facebook group. There was a total of 308 posts, 355 comments, and 2,437 reactions,	I

		including Likes, comments, shares, over the 10-week program duration. The busiest weekdays in terms of user activity were Mondays, which immediately followed kit releases, then Wednesdays. The types of posts that generated the most reactions were “Wellness Wednesday” check-ins, where group members were asked to respond to questions such as how they were feeling that day or to share a positive change in their life that they hoped to sustain beyond the pandemic. Also popular were profiles of project partners that invited questions from group members, such as an expert in special education for young children.	
25	Active-duty	Emotional distress in one parent was significantly associated with distress in the other. Greater baseline distress in both M and civilian par- ents was associated with greater positive change in their own Family Assessment Device (FAD) scores. Positive change in the FAD for M par- ents was significantly associated with positive change in the FAD for the civilian parent. In addition, a positive change in FAD for the M parent was associated with reduced distress for both the M parent and the civilian parent. Further, a positive change in FAD for the M parent was associated with higher impact scores for both M parents and the civilian parents. A positive change in the FAD for civilian parents was associated with their own reduced distress.	II
26	Active-duty	The program facilitated prenatal and postpartum maternal role adaptation. Immediately after the intervention, the participants in the experimental group reported greater prenatal adaptation than the control group in the “fear of pain or loss of self-control and self-esteem”, “preparation for labor,” and “concern for the wellbeing of self and baby”, indicating that the women who participated in the intervention felt better prepared to handle the challenges related to labour and more confident in their ability to deal with labour events than the women who participated in the control group. They also were less likely to dwell on possible negative pregnancy and birth outcomes. Significant group differences were found immediately after the intervention (completion) in internal resources with a large effect size in experimental group mothers compared to control group mothers. No statistically significant group differences were found between groups in internal resources at baseline or 6 weeks postpartum. Further, external resources showed no statistically significant differences at baseline between the be- tween the groups. At completion, the experimental group mothers reported greater perceived social support than the control group, with a medium effect size, yet not statistically significant at 6 weeks postpartum. Attrition: About ¼ attritted with most failing to complete all 3 data collection points, and 3 were removed for medical complications. There were no significant differences in participant attrition by group.	I
27	Reintegration	The program was a feasible adaptation of the Multifamily Group model for V and their families. Satisfaction: (1) 60% believed mental health care was excellent and 37% believed mental health care was good; (2) 71% stated they would definitely recommend REACH to others and 28% responded “Yes, I think so”; (3) 51% stated that REACHED somewhat helped them to deal with problems more effectively and 47% said it helped a great deal; (4) 60% stated they were very satisfied with the REACH program and 39% said they were mostly satisfied; (5) 85% were very satisfied with their therapist and 14% were mostly satisfied. Further, participants reported	II

		that they liked learning about the diagnoses, which improved their understanding of their family members, and helped them discover that they were not alone. The most common com-plaints with the program were that the Phase II sessions were too short (n = 25, 12%), having to travel to the hospital for the REACH classes (n= 12, 6%), or having too few individual family sessions (n = 9, 4%). Attrition: Majority completed Phase I and Phase II. About 1/3 attritted be- tween the start of Phase I and the start of Phase II. Given the long-term nature of REACH, reminder letters and day-before-appointment phone calls and attendance awards at the end of Phase II and III, such as mugs or pens were applied.	
28	Reintegration	Demographic characteristics of participants' M or V do not differ between online and in-person programs. The majority of participants re- ported that the program was relevant and helpful, and all participants would recommend it to other MF. Participants experienced improvement in stress, depressive symptoms, and self-efficacy. Online participants were more than double the number of in-person participants. Two-thirds found that six sessions were just right, whereas the rest reported wanting more sessions. Most participants reported that 90 minutes per session was optimal. Only 38 participants completed the qualitative feedback survey.	II
29	Pre-deployment active-duty/post- deployment	The program decreased anxiety in expecting women for Identification with a Motherhood Role and Preparation for Labor, particularly in participants whose spouses were deployed or going to be deployed. Partnered women had less prenatal anxiety, yet partner deployment at any time during the prenatal period significantly increased pregnancy-specific anxiety. Mothers who had a high school diploma experienced the greatest anxiety for Acceptance of Pregnancy and those with some college had the greatest protective effect. Conversely, for prenatal anxiety related to Well-Being of Self and Baby in Labor, those with a high school diploma had the least anxiety, and college graduates had the greatest anxiety. Anxiety increased with maternal age. Resilience outcomes improved for both groups, so either care condition was likely helpful. No significant changes were noted in participant depression symptoms. Of note, the overall group mean for depression symptoms was low at baseline. No covariates were significant within the final adjusted model. Self-esteem did not increase significantly for participants. Being un- married/not partnered and having higher education were significant predictors of resilience. Unmarried participants had mean resilience scores greater than those of the married participants, and pregnancy and some college presented a protective effect. Attrition: About 1/3 attritted.	I
30	Active-duty/post-deployment, reintegration	Hypothesis 1: There was a significant increase in knowledge of the 7 Cs of Resilience between baseline and completion, and between baseline and 6 months, where 38 % identified most of the 7 Cs correctly. Hypothesis 2: There was also a significant increase in knowledge of the reintegration of military family members between baseline and completion, and between baseline and 6 months. Hypothesis 3: There was a significant effect of time on participants' awareness of the current needs of military teens in their community. Attrition: While the majority completed pre and post tests, only 1/3 completed all three timepoints.	II

31	Post-deployment	Parents felt that their children had more behaviours in military families than civilian families, and older children/teens felt they had more behaviours during their parent deployment and learned some skills related to managing their behaviours. Reported total difficulties with child behaviour in MF was significantly higher than the National Health Interview Survey for the civilian sample. Children. At least 3/4 of teens responded ‘‘really true’’ or ‘‘kind of true’’ to missing the deployed parent, feeling like they had to help more, and worrying about the non- deployed parent, whereas between 1/2 and 2/3did so about being upset at not knowing when the deployed parent would return, having to do a lot more house chores, and fighting more with siblings. For the six items on new ideas for talking to their parents, ¾ to the majority of teens responded either ‘‘yes’’ or ‘‘sort of ’’. A total ‘‘got new ideas’’ cumulative score was created with the teen’s six responses, ranging from 0-12, rendering ¾ who were above the midpoint, 7 or higher. Program fidelity was reportedly good overall. The PTS program was ineffective for children 3-6 years of age, as they did not understand the contents and their shorter attention spans interfered with their activity completion or engagement in debriefing.	II
32	Post-deployment	Spouses found the information helpful overall, but only half had implemented the information. Further, only those who had continued family supports had sustained improvements at follow-up and reported using information provided. Overall ratings of the seminar on the first client satisfaction form were positive, with scores ranging from 3.9 on 5 for the module on Financial Preparation to 4.7 for the module on Flexible Marriage. The global rating of the program was high, 4.8. At follow-up, some participants indicated that they valued feeling less alone and that their experience of separations had been normalized. A few participants indicated that they realised the importance of communication in the marriage, and a few more indicated that they learned how to cope better with separation stress. The program might have helped over ½ the participants during deployment either “a lot” or “quite a lot”. 2/3 of participants indicated that they had made most or all of the intended changes, or a few, one, or none of the changes. Significant improvement also widely varied; group 1 improved in 22 of the 35 variables, in comparison with only 1 improvement in Group 2. Significant deterioration was found in Group 2. This suggested that those who incorporated the principles of separation resilience in their families showed relative improvements in most factors assessed. Attrition: Under ¾ completed pre and post testing only.	II

1: **Level I (one):** Evidence from at least one properly randomized, controlled trial; **Level II (two):** Evidence from at least one well-designed clinical trial without randomization, from cohort or case- 146 control analytic studies (preferably from more than one centre), from multiple time-series studies or from dramatic results in uncontrolled experiments; **Level III (three):** Evidence from opinions of 147 respected authorities based on clinical experience, descriptive studies or reports ofexpert committees.

[Adapted from the Government of Canada, 2016: Levels and quality of evidence. Available online: https://www.canada.ca/en/public-health/services/infectious-diseases/sexual-health-sexually-transmitted-infections/canadian-guidelines/levels-quality-evidence.html (accessed on April 13, 2023)].

Table S5. Brief Description of the 22 Programs or Interventions Found in This Review.

Identifier Total articles	Program or Intervention Name	Program or Intervention Description
9, 10, 23<br>3 articles	*After Deployment, Adaptive Parenting Tools* (ADAPT)	An adaptation of the PMTO parenting program, ADAPT is a 14-week group-based resilience-building intervention for military families that was designed to be convenient for military families (e.g, facilitators are military professionals, delivery sites are near participant homes). The ADAPT intervention principally aims to help military parents learn parenting skills like (1) emotion regulation, (2) teaching about emotions, discipline, and teaching with encouragement, ultimately to help military parents respond effectively to their children's emotions. The ADAPT curriculum covers content such as coping, emotion-regulation, identifying emotions, mindfulness, positive parenting practices, and family strategies to manage stressors.
26<br>1 article	*Baby Boot Camp* (BBC)	BBC is a 4-week intervention for pregnant military wives to help them adapt to their new maternal role. Each week of the BBC program has a distinct intervention focus. The first week focuses on identifying stressors of motherhood and military life. The second week focuses on identifying internal strengths and identifying ways that participants can apply them in daily life. The third week focuses on shifting to external resources in one's family and community. The fourth week focuses on role-playing, group conversations, and preparation for future challenges. Each week contains a blend of psychoeducation, activities, and informational handouts.
20, 21<br>2 articles also on RIRO	*Bounce Back and Thrive!* (BBT)	*The Bounce Back and Thrive!* (BBT) is a group-based psychoeducational resilience program for families. Grounded in the evidence-base of the Penn Resiliency Program, BBT is designed for parents who have children between the ages of birth to 8 years. The program is divided into two phases: *Adult Skills* and *Child Application Skills*. The ultimate goal of this program is to help parents build resilience skills, so they can learn how to model and teach those skills to their children.
2<br>1 article	*Coming Home Project* (CHP)	CHP is a post-deployment reintegration program that provides 4-day military-family retreats for Veterans, service members, and their families, as well as service providers who serve these populations. Retreats comprise psychoeducational and skill trainings (e.g., mindfulness, family skills), group activities, support and discussion groups, and reserved free time for individuals and families. At the end of each retreat, participants are given resources to use in their own communities.
8, 17-19, 25<br>5 articles	*Families Overcoming Under Stress* (FOCUS)	Adapted from three other resilience-building interventions, FOCUS is an intervention designed to help improve military family dynamics, including parent-child and parent-parent interactions and relationships. FOCUS is delivered over eight sessions, on parent only, parent-child, child-only, and whole family sessions that last one to one and a half hours. Sessions incorporate psychoeducation, narrative activities, resilience skill practice, and other intrafamily dynamic activities.

		FOCUS skill-building groups (SBG's) is implemented in school settings to teach resilience skills in nine sessions to help military children or youth cope with stressors stemming from military life.
24<br><br>1 article	*Families Tackling Tough Times Together* (FT)	FT is a 10-week family resilience intervention adapted from the WASH Family Resilience framework that aims to help families endure hardships, and to grow from such hardships that facilitates improved future adaptation. The modules focus on three broad family-life domains: shared beliefs, communication/problem-solving, and organization.
22<br><br>1 article	*Little Children, Big Challenges: General Resilience* (LCBC)	LCBC is a digital multimedia program designed for children. The purpose of this intervention is to improve children's coping and socio-emotional abilities at home and school, including teacher-child relationships.
29<br><br>1 article	*Mentors Offering Maternal Support* (MOMS)	MOMS is a social support program designed for active duty military women and civilian wives of service members to help reduce their pregnancy-related anxieties that are associated with low birth weight and preterm births. Ultimately, the program aims to provide participants with social support within military communities to help decrease their anxieties.
7<br><br>1 article	*Military Family Foundations* (MFF)	The MFF program is an adaptation of the *Family Foundations* online program, specifically for military families. The content of MFF focuses on improving cohesiveness and adjustment between partners during the transition to parenthood, both before and into parenthood, including a blend of audio and visual media.
1<br><br>1 article	*Military Teen Adventure Camp* (MTAC)	MTAC is a five-day camp designed to help military service members that have recently returned from deployment to reconnect with their family, specifically their teens. The camp incorporates experiential outdoor problem-solving activities for parents and their teens with the goal of increasing cooperation and connection within the family.
30<br><br>1 article	*Military Teen Ambassador's* (MTA)	The *Military Teen Ambassadors* (MTA) program was developed by the Boys and Girls Clubs of America (BGCA) and the United States Armed Forces. This program applies the 7 C's model of resilience to promote teen sense of agency and develop a voice, ultimately to build teen leadership and resilience skills, with the long-term goal of helping them become positive change agents in their communities.
15<br><br>1 article	*Mind Body Awareness Program* (MBA)	MBA is adapted for military teens from a mindfulness program designed to address youth delinquency and violence. The five modules comprise an introduction to mindfulness, mindfulness of the body, emotional awareness and empathy building, the matter of choice, and change and interconnectedness.
4, 13<br><br>2 articles	*Mission Reconnect* (MR)	MR is a self-directed multimedia intervention designed for Veterans and their partners. This intervention offers a combination of video, audio, and handout resources for participants. MR is grounded in a biopsychosocial health framework with exercises or techniques to address physical, social, and psychological realms.

31<br><br>1 article	*Passport Towards Success* (PTS)	PTS is a program based on an "island theme" designed to help children improve resilience. Children rotate between three islands that last about 45 to 75 minutes: (1) feelings island, (2) relaxation island, and (3) communication island. For example, skills taught are island-specific, such as listening skills on the communication island. The program also comprises interactive practice opportunities, and debriefings with program facilitators, followed by taking a poster home to encourage reciprocal family talk.
20, 21<br><br>2 articles also on BBT	*Reaching In… Reaching Out* (RIRO)	*Reaching In… Reaching Out* (RIRO) is a trauma- and evidence-informed resilience program for families, adapted from the University of Pennsylvania's Resiliency Program. RIRO is divided in two parts: (1) building resilience skills such as emotion regulation and cognitive skills, and (2) teaching parents how they can apply and teach these resilience skills to their children.
27<br><br>1 article	*Reaching out to Educate and Assist Caring, Healthy Families* (REACH)	REACH is an intervention adapted from Multifamily Group Programs (MFG) for Veterans, considering their affective disorders, PTSD, as well as their families. REACH is composed of three phases delivered over nine months. The first phase includes four 50-minute sessions; both Veterans and their families are present. The second phase offers group sessions and psychoeducational seminars also involving both Veterans and their families. Sessions are facilitated by psychologists trained in delivering REACH. The third phase concentrates on problem-solving, which varies by group.
14<br><br>1 article	*REBOOT Combat Recovery*	REBOOT is a 12-week Christian recovery program for military members with combat-based trauma. The program is peer-led and grounded in three theoretical models on health, social learning, and occupational therapy practice. Using faith-based concepts, REBOOT aims to foster trusting relationships and reframe adversity as opportunities for post-traumatic growth and life purpose.
32<br><br>1 article	*Separation Resilience Seminar* (SRS)	SRS is a one-day psychoeducational seminar for couples with one partner enlisted in the military, specifically the navy. The seminar introduces topics such as psychoeducation about schizophrenia, fostering healthy family beliefs, and teaching adaptive family coping strategies to navigate routine separations.
3, 28<br><br>2 articles	*Stress Management and Resiliency Training: Relaxation Response Resiliency Program* (SMART-3RP))	SMART-3RP is an eight-week resilience group intervention that includes meditation, imagery, and breathing exercises. The format is flexible, allowing participants to choose activities that fit them most, ultimately to manage stress.
6, 11, 12<br><br>3 articles	*Strong Military Families* (SMF)	SMF is a 10-week resilience intervention adapted from Mom Power, which is for civilians. SMF is designed for military families with children. This intervention aims to help parents learn positive parenting and self-care to facilitate family reunification and reconnection post-deployment. The intervention is delivered either at home in a psychoeducational format or in a multifamily group forma. In the home-based psychoeducational format, families are provided

		psychoeducational materials that cover the same core content as the multifamily group, without social interaction with other participants or facilitators. In the multifamily group format, parents meet as a group for 10 sessions, plus about 2 or 3 sessions as a couple with facilitators, while their children engage in play group activities. Sessions are designed to help parents understand their child's behaviour, ultimately for positive and nurturing parenting. .
5<br><br>1 article	*Tell Me A Story* (TMAS)	TMAS isa bibliotherapy intervention that focuses on developing resilience from a positive psychology framework and is designed to help build resilience in military children by facilitating discussions between parents and their children about resilience-related topics. Bibliotherapy resilience interventions are typically delivered in a four-step format: (1) choosing the material to be read (pre- reading), (2) reading together, (3) post-reading discussion(s), and (4) engaging in an activity to reinforce the lessons
16<br><br>1 article	*Thank You, Sorry, Love* (TSL)	TSL is a 10-session program that includes, Thank you (1–4 sessions), Sorry (5–7 sessions), and Love you (8–10 sessions). Participants practice tasks from recall, recognition, realization, reinforcement, and recharging (5Re mechanism). Tasks centre mainly on the fam- ily system. Participants who have practiced TSL expressions and behaviours with their family were asked to expand their practice to community members such as peers and teachers.

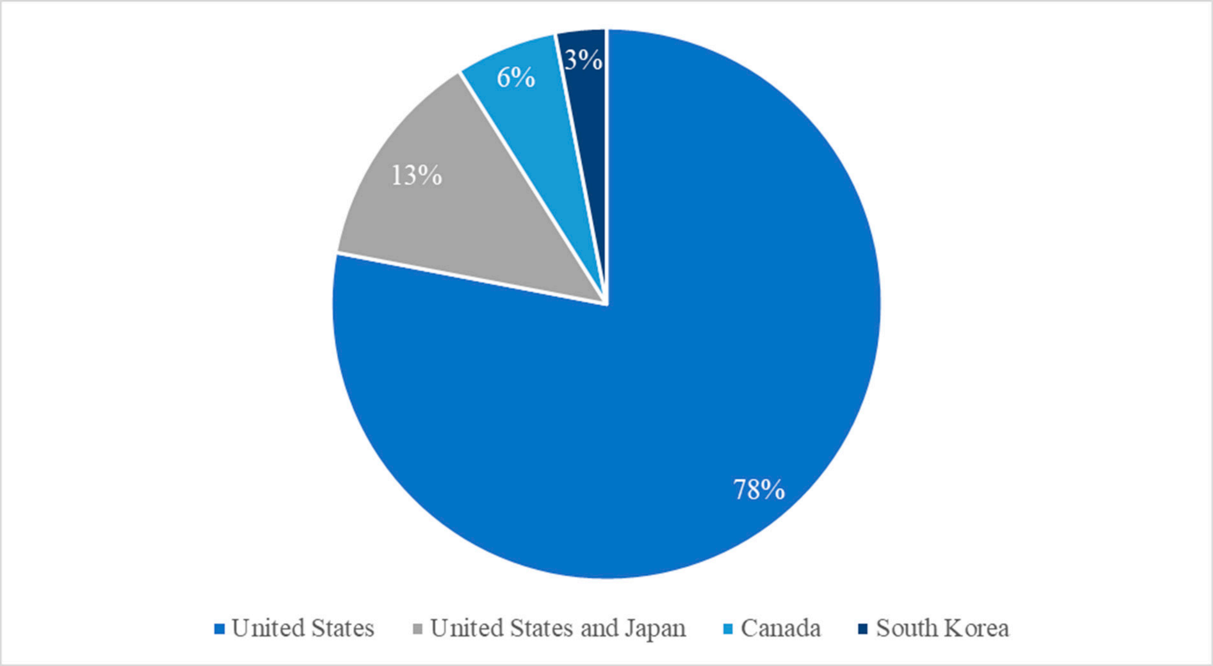

Figure S1. Country of Each Study.
United States: 1- 15, 22-24, 26-32
United States and Japan: 17-19, 25
Canada: 20, 21
South Korea: 16

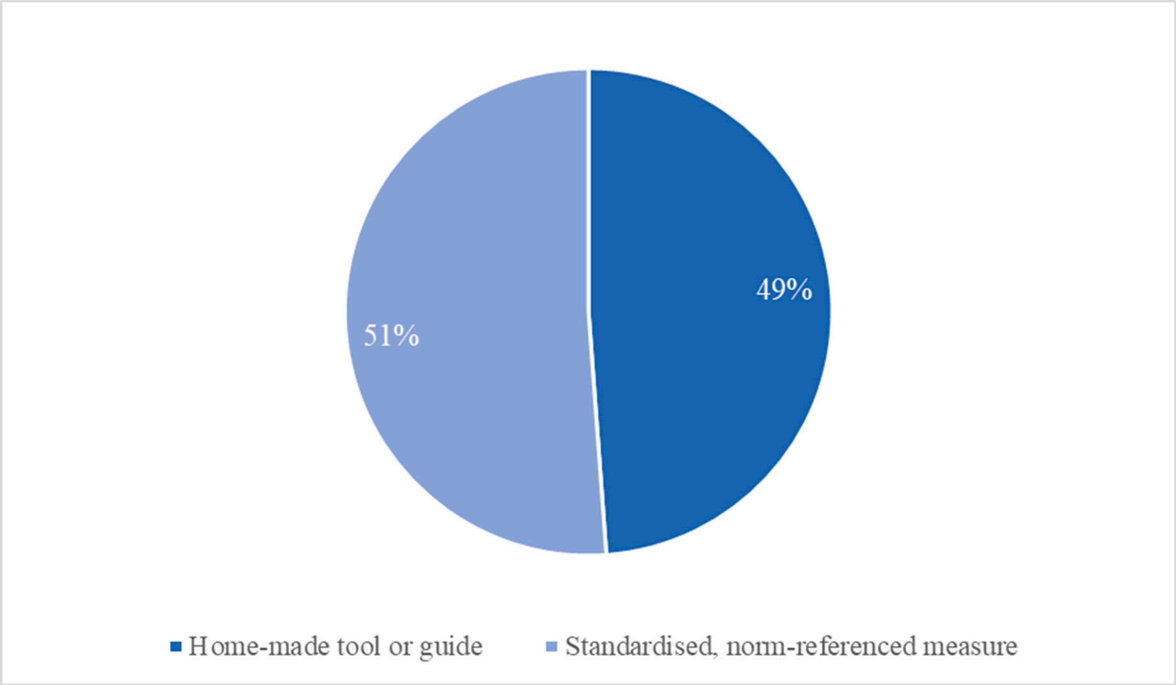

Figure S2: Outcome Tools or Measures Used
Home-made tool or guide: 1-6, 8, 11-13, 15, 20-24, 27, 30-32
Standardised, norm-referenced measure: 1, 4-7, 10, 11, 13, 14, 16-19, 22, 26-32
